# Study on the Preparation and Test Method of Transformer Oil Used in Laboratory

**DOI:** 10.3390/ma17236010

**Published:** 2024-12-09

**Authors:** Zeming Sun, Minxia Shi

**Affiliations:** 1 China Airport Planning & Design Institute Co., Ltd., Northwest Branch, Xi’an 710075, China; 2 Key Laboratory of Ultra-Weak Magnetic Field Measurement Technology, Ministry of Education, School of Instrumentation Science and Optoelectronics Engineering, Beihang University, Beijing 100191, China

**Keywords:** transformer oil, cellulose particle, laboratory, test method

## Abstract

The power transformer is one of the most important parts of a power system. The transformer oil in a transformer not only increases its insulation strength, but also helps its cooling. Cellulose particles are one of the main factors affecting the breakdown characteristics of transformer oil, and the withstand voltage test can effectively detect the quality of transformer oil. Therefore, the withstand voltage test on transformer oil with different cellulose particle content levels in the laboratory can determine the breakdown characteristics of transformer oil and the method of improving the insulation strength of power transformers. However, there is lack of a set of effective preparation and test methods of transformer oil for laboratory use in the industry. Based on national standards and engineering practice, this paper puts forward a method of preparing transformer oil with different cellulose particle content levels in the laboratory and a method of the withstand voltage test on transformer oil in the laboratory, which can improve the efficiency and the scientificity of such experiments.

## 1. Introduction

The power transformer is one of the most important parts of a power system; it plays the role of connecting transmission lines of different voltage levels. Therefore, the normal operation of the power transformer determines the safety and stability of the power system [1]. At present, the power transformer used in China is mainly the oil-immersed transformer, and its main insulation structure is an oil–paper composite insulation structure. The oil in a transformer not only increases its insulation strength, but also helps its cooling [2]. The transformer oil in operation has many impurity particles, such as dust particles brought by installation, metal particles dropped from the shell, and carbon particles generated by electrochemistry.

Engineering practices show that the main impurity particles inside the oil-immersed transformer mainly come from aged insulating cardboard, and the cellulose particles shed from the insulating cardboard will mix with the transformer oil and move with the electric field [3,4]. When the power transformer is running, if there are cellulose particles in the transformer oil, these cellulose particles will move to the high electric field region under the action of the electric field. And a large number of cellulose particles gathered in the electrode region will form a “cellulose bridge”, which may cause partial discharge or even insulation breakdown of the power transformer [5,6].

The withstand voltage test can effectively detect the quality of transformer oil, and the power frequency voltage test and the lightning impulse voltage test are routine test items of a power transformer. In order to reduce the volume of the test device and improve the test efficiency, the relevant research of the oscillation lightning impulse voltage test on the power transformer is also gradually carried out in engineering practice [7,8,9]. Therefore, it is very important to study the effect of cellulose particles on the insulation properties of transformer oil and the withstand voltage test method on transformer oil.

However, there is no general preparation and test method of transformer oil used in the laboratory, and the level of cellulose particles in transformer oil samples varies from person to person, which reduces the scientificity of transformer oil testing in the laboratory [10,11,12,13,14,15]. This paper first studies the method of preparing transformer oil with different cellulose particle content levels in the laboratory, then studies the construction of the withstand voltage test system in the laboratory, and finally studies how to use the system to test the transformer oil, which can improve the efficiency and the scientificity of such experiments.

## 2. Preparation of Transformer Oil Used in Laboratory

### 2.1. Standard-Control Transformer Oil Sample

When studying the effect of cellulose particle content level on the breakdown characteristics of transformer oil, we need to add cellulose particles in pure transformer oil to prepare transformer oil samples with different cellulose particle content levels. Therefore, it is necessary to pre-treat the transformer oil from the factory to avoid the influence of oil quality on the test results.

In this paper, the KI-25X transformer oil (produced in Karamay Oilfield, Xinjiang, China) was selected as the sample material; it is widely used in oil-immersed transformers in China. Because the physicochemical properties of KI-25X transformer oil meet the relevant requirements of the national standard “Guide to the Choice of Power Transformer Insulating Oil (DL/T 1094-2018)”, KI-25X can meet the research needs [16].

When taking transformer oil samples, this paper refers to the requirements of national standards “Method for Manual Sampling of Petroleum Liquids (GB/T 4756-2015)” and “Method of Sampling for Transformer and Turbine Oils in Electric Power Industry (GB/T 7597-2007)”, which reduces the pollution of transformer oil samples caused by manual operation [17,18].

During pretreatment, the KI-25X transformer oil is filtered twice through a 1 μm filter membrane, and then dried in a vacuum drying oven for 48 h. When drying, the temperature in the vacuum drying oven is 80 °C, and the air pressure is less than 5 kPa, so the boiling point of water is 29 °C, but the boiling point of transformer oil is greater than 90 °C. When filtering small amounts of transformer oil in the laboratory, a complete set of vacuum sand core solvent filter devices can be used.

The quality parameters of the transformer oil sample after pretreatment are shown in Table 1, and the transformer oil sample meets the requirements of 500 kV voltage grade transformers. The quality indicators in Table 1 select the relatively strict quality indicators in national standards “Fluids for Electrotechnical Applications—Unused Mineral Insulating Oils for Transformers and Switchgear (GB 2536-2011)”, “Quality of Transformer Oils in Service (GB/T 7595-2017)”, and “Limited Value of Particulate Pollutant of Transformer Oil (DL/T 1096-2018)” [19,20,21].

**Figure 1:**
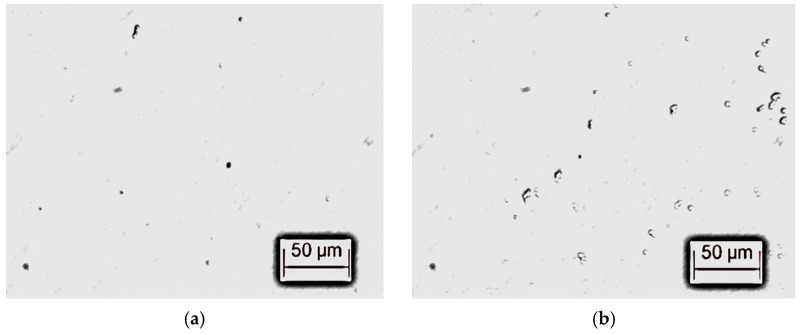
Microscopic photograph of impurity particles in transformer oil after pretreatment. (**a**) Impurity particle morphology 1. (**b**) Impurity particle morphology 2.

After pretreatment, the transformer oil can be used in the power industry, but there are still impurity particles in the transformer oil (Figure 1 shows a microscopic photograph of impurity particles). These impurity particles are introduced due to the manufacturing process and the use of the transformer oil, and cannot be removed by physical means. Therefore, this paper refers to the pretreated transformer oil as the “Standard-control transformer oil sample”, which is the control group of the test. And the test results of the “Standard-control transformer oil sample” represent the physicochemical properties of industrial transformer oil.

In this paper, cellulose particles are added to the “Standard-control transformer oil sample” to prepare transformer oil with different cellulose particle content levels, and this paper refers to the contaminated transformer oil as the “Test-control transformer oil sample”. And the test results of the “Test-control transformer oil sample” represent the effect of cellulose particle content level on the physicochemical properties of transformer oil.

### 2.2. Test-Control Transformer Oil Sample

#### 2.2.1. Selection and Treatment of Cellulose Particles

Microcrystalline cellulose is prepared from natural cellulose by diluting acid hydrolysis to the ultimate polymerization degree. Its appearance is white powder, it is insoluble in water and organic solvents, and the particle size is less than 100 μm. It is a kind of pure cellulose depolymerization product, which is widely used in medicine, food, cosmetics and light chemical industries [22].

According to the detection indicator of the national standard “Determination of Particulate Contamination in Oil Used in Power Industry (DL/T 432-2018)” and the actual situation of the cellulose impurities in the transformer oil, the microcrystalline cellulose particles have the same molecular structure and the same size as the cellulose impurities in the transformer oil, so it is a suitable cellulose particle to prepare the “Test-control transformer oil sample” [23].

Therefore, this paper uses microcrystalline cellulose particles (medical grade, produced in Yongshun Chemical, Yongshun, China) to simulate the actual cellulose particles in transformer oil, and uses microcrystalline cellulose particles to prepare transformer oil samples with different cellulose particle content levels. The cellulose particles mentioned below are all microcrystalline cellulose particles.

**Figure 2:**
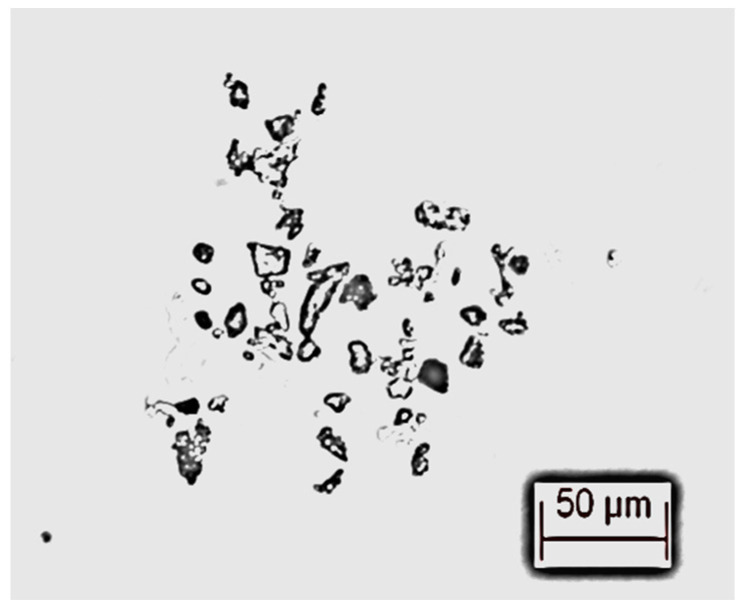
Microscopic photograph of microcrystalline cellulose.

The microscopic photographs of microcrystalline cellulose particles were taken using a Leica-DMI 3000B inverted fluorescence microscope, and the morphology of microcrystalline cellulose particles under the microscope is shown in Figure 2. Compared with the self-contained impurity particles of transformer oil in Figure 1, the morphology of microcrystalline cellulose particles is more diverse and the particle diameter is larger, which can distinguish them from the self-contained impurity particles of transformer oil.

Microcrystalline cellulose has water absorption, and it is easy to aggregate to form large particles after absorbing water, which may affect the test results. In order to make the shape of microcrystalline cellulose particles more similar to the actual cellulose impurity particles in transformer oil, the microcrystalline cellulose particles in this paper are dried in a vacuum drying oven for 12 h, and then the particles larger than 100 μm in diameter are screened with a sampling screen. When drying, the temperature in the vacuum drying oven is 80 °C, and the air pressure is less than 5 kPa.

After treatment, the particle size distribution of microcrystalline cellulose particles is uniform and conforms to reality. Although microcrystalline cellulose will be polymerized in transformer oil during use, the size and quantity after polymerization are also consistent with the actual particles in transformer oil.

#### 2.2.2. Determination of the Cellulose Particle Content Level in Transformer Oil Samples

The results show that the impurity particles with larger diameter have greater influence on the breakdown characteristics of transformer oil, and it is necessary to pay attention to the particles with a diameter larger than 5 μm in the test [24,25,26]. Therefore, the cellulose particles in the “Test-control transformer oil sample” are the main factors affecting the breakdown characteristics of transformer oil, that is, the transformer oil samples prepared with microcrystalline cellulose particles meet the needs of the withstand voltage test of transformer oil, and the test results can show the effect of cellulose particle content levels on the breakdown characteristics of transformer oil.

In order to study the effect of cellulose particle content levels on the breakdown characteristics of transformer oil, transformer oil samples with different concentrations of cellulose particles need to be prepared for testing. Because particles in transformer oil with a diameter greater than 100 μm are mainly affected by gravity rather than electric field forces, the particulate pollution level of transformer oil during normal operation of a power transformer is generally determined by small particles, especially particles with a diameter of 5 μm [24,25,26]. Therefore, four kinds of transformer oil samples are prepared in the laboratory based on the number of impurity particles with particle diameter of 5–15 μm per 100 mL of transformer oil in this paper, according to the classification index of particulate pollution in transformer oil in the CIGRE standard “Brochure 157” [23,27].

(a) Standard-control transformer oil sample

No cellulose particles are added to the transformer oil sample, which is the control group in experiments. The number of impurity particles with particle diameter of 5–15 μm is about 10^3^ per 100 mL.

(b) Low-pollution transformer oil sample. The number of cellulose particles with particle diameter of 5–15 μm is about 10^4^ per 100 mL.
(c) Middle-pollution transformer oil sample. The number of cellulose particles with particle diameter of 5–15 μm is about 10^5^ per 100 mL.
(d) High-pollution transformer oil sample. The number of cellulose particles with particle diameter of 5–15 μm is about 10^6^ per 100 mL.

#### 2.2.3. Preparation of Transformer Oil Samples

In order to facilitate the storage and use of transformer oil samples, a high-borosilicate reagent bottle with the volume of 500 mL was selected as the oil container. The reagent bottle (including the cap) is washed with detergent before use, and then washed twice with alcohol (analytical reagent, produced in Sinopharm Chemical, Nanjing, China). If there is still oil in the reagent bottle, it needs to be washed with acetone (analytical reagent, produced in Sinopharm Chemical, China), and the experimenter should operate in the fume hood to avoid poisoning.

(a) Low-pollution transformer oil sample

Firstly, 3000 mL of pretreated transformer oil and 7.8 mg of treated microcrystalline cellulose particles are added into the conical flask. Then, a magnetic mixer is used to stir the transformer oil for 5 min to make the cellulose particles evenly distributed in the transformer oil. Finally, the transformer oil is divided into reagent bottles at 500 mL per part.

In this paper, the magnetic mixer and its stirrer were selected according to the national standard “Insulating Liquids—Determination of the Breakdown Voltage at Power Frequency (GB/T 507-2002)”. The length of the stirrer is 25 mm, the diameter of the stirrer is 8 mm, and the stirring speed of the magnetic mixer is between 250 and 300 r/min [28].

(b) Middle-pollution transformer oil sample

A total of 500 mL of pretreated transformer oil and 13 mg of treated microcrystalline cellulose particles are added to the reagent bottle.

(c) High-pollution transformer oil sample

A total of 500 mL of pretreated transformer oil and 130 mg of treated microcrystalline cellulose particles are added to the reagent bottle.

**Figure 3:**
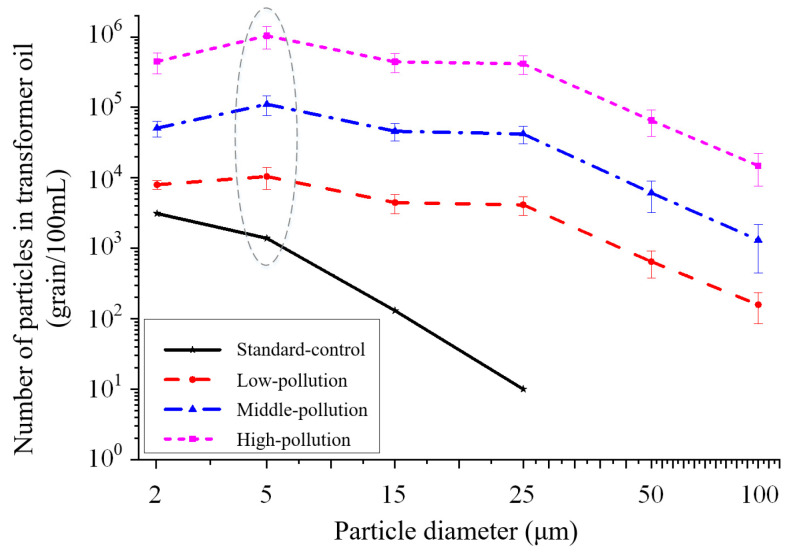
Distribution of particle numbers in transformer oil samples.

The four transformer oil samples used in this paper were detected according to the national standard “Determination of Particulate Contamination in Oil Used in Power Industry (DL/T 432-2018)”, and the detection results in Figure 3 show that the distribution of particles in the transformer oil meets the experimental requirements and is consistent with the actual working conditions of a transformer.

The national standard “DL/T 432-2018” takes the particle diameter range as the detection index, and the interval value is represented by the left endpoint of the interval. That is, the number of particles with a particle diameter of 2 μm, 5 μm, 15 μm, 25 μm, 50 μm, 100 μm in Figure 3 indicates the number of particles with a particle diameter range of 2–5 μm, 5–15 μm, 15–25 μm, 25–50 μm, 50–100 μm, and above 100 μm.

### 2.3. Sedimentation Analysis of Cellulose Particles in Transformer Oil Samples

The prepared transformer oil samples are evenly distributed suspensions, but after a period of time, the cellulose particles will settle down due to gravity. Gradually, the particles on the upper and lower liquid surface of the transformer oil sample are not consistent, so it is necessary to stir the transformer oil sample during the experiment.

**Figure 4:**
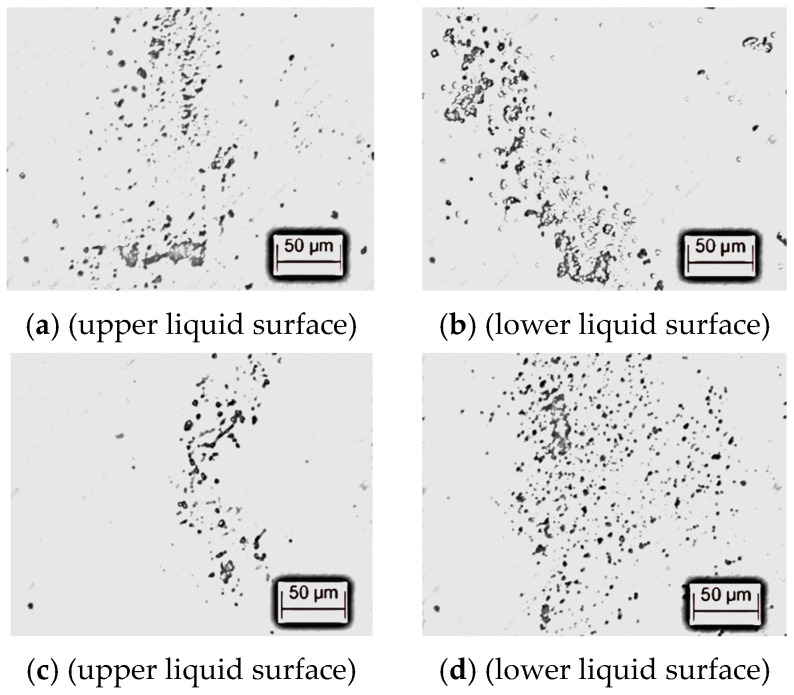
Distribution of impurity particles in the “High-pollution transformer oil sample”. (**a**) Standing for 8 min. (**b**) Standing for 8 min. (**c**) Standing for 15 min. (**d**) Standing for 15 min.

In this paper, a Leica-DMI 3000B inverted fluorescence microscope was used to observe the “High-pollution transformer oil sample”, and Figure 4 shows the distribution of cellulose particles in the transformer oil.

After stirring and standing for 8 min, the cellulose particles in the upper and lower liquid surface of the “High-pollution transformer oil sample” are equivalent, that is, the cellulose particles in the transformer oil are evenly distributed, and the sample meets the test requirements.

After stirring and standing for 15 min, the concentration of cellulose particles in the lower liquid surface of the transformer oil sample is much greater than that in the upper liquid surface, so it is necessary to control the static time of the transformer oil sample for less than 8 min during the test.

## 3. Construction of the Withstand Voltage Test System in Laboratory

### 3.1. Laboratory Withstand Voltage Test Platform

The withstand voltage test of the transformer oil sample includes the power frequency voltage test, lightning impulse voltage test and oscillation lightning impulse voltage test. And the laboratory power frequency voltage test platform is relatively mature and it is easy to buy on the market.

**Figure 5:**
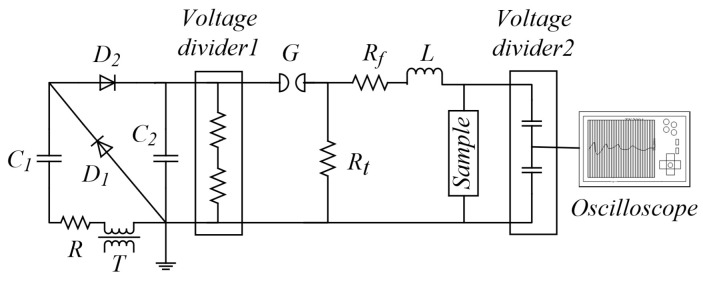
Circuit diagram of the laboratory impulse voltage test platform.

Based on engineering practice, this paper provides the following laboratory impulse voltage test platform, and its circuit is shown in Figure 5. It can generate a lightning impulse voltage or oscillation lightning impulse voltage by replacing a small amount of electrical components. And Table 2 shows the circuit parameters of the platform to generate the standard lightning impulse voltage (1.2 ± 30/50 ± 20% μs) and the oscillation lightning impulse voltage (310 kHz, 1.2 ± 30/50 ± 20% μs).

The laboratory impulse voltage test platform in this paper is charged by a voltage doubling circuit to the main capacitor C_2_, the resistance R in the circuit is the current-limiting resistance, and the ball gap G is photoelectrically triggered to ensure safe operation.

Voltage divider 1 is used to measure the charging voltage of the impulse voltage generator, and voltage divider 2 is used to measure the actual voltage applied to the transformer oil sample.

The output waveform of the impulse voltage can be controlled by adjusting the resistance Rf and Rt, and the inductor L should be added when the platform generates oscillation lightning impulse voltage.

**Figure 6:**
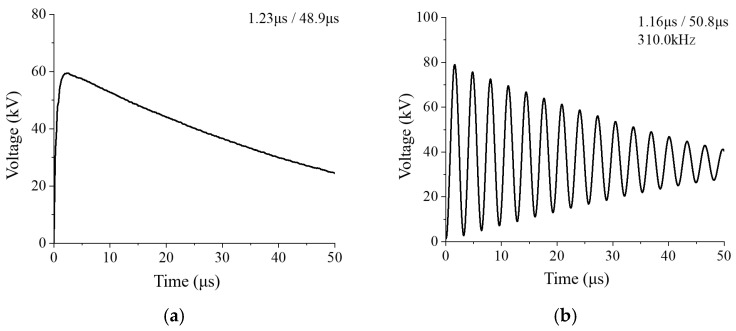
Measured waveform of the laboratory impulse voltage test platform. (**a**) Measured waveform of standard lightning impulse voltage. (**b**) Measured waveform of oscillation lightning impulse voltage.

The maximum output voltage of the test platform is 100 kV, and the output waveform shown in Figure 6 can meet the experimental requirements.

### 3.2. Container of the Transformer Oil Sample

The transformer oil sample used in this paper needs to be packed in a customized container. The customized container should not only store the transformer oil sample without pollution, but also form the electric field needed for the experiment between the electrodes.

**Figure 7:**
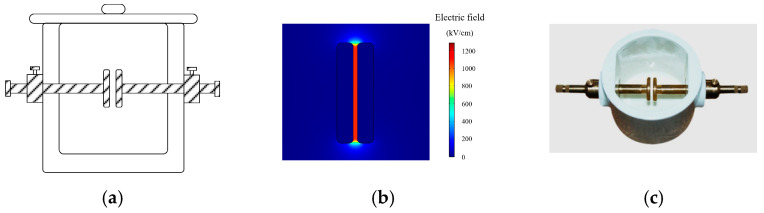
Uniform electric field test container. (**a**) Structure of the uniform electric field test container. (**b**) Electric field distribution between the plate-plate electrodes. (**c**) Photograph of the test container.

Because there is no explicit standard for the lightning impulse voltage test in a uniform electric field, this paper designed the uniform electric field test container according to relevant research and the national standard “Insulating Liquids—Determination of the Breakdown Voltage at Power Frequency (GB/T 507-2002)”. The customized container is shown in Figure 7a, which can form the uniform electric field between the plate-plate electrodes [28,29]. And the container adopts a closed structure to avoid the pollution of the sample in tests.

**Figure 8:**
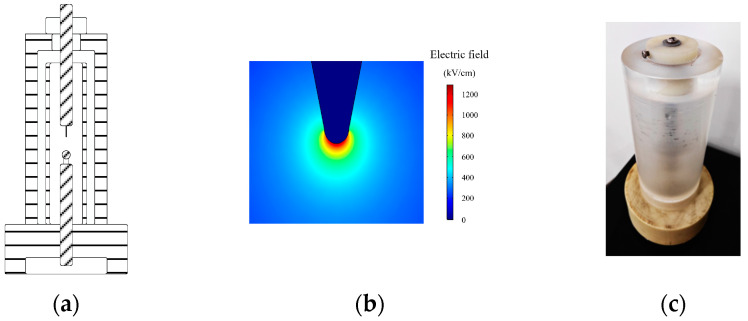
Uneven electric field test container. (**a**) Structure of the uneven electric field test container. (**b**) Electric field distribution between the needle-ball electrodes. (**c**) Photograph of the test container.

For the lightning impulse voltage test in an uneven electric field, this paper designed the uneven electric field test container according to the national standard “Methods for the Determination of the Lightning Impulse Breakdown Voltage of Insulating Liquids (GB/T 21222-2007)”. The customized container is shown in Figure 8a. It can form an uneven electric field between the needle-ball electrodes [30]. And the container adopts a closed structure to avoid the pollution of the sample in tests.

The electrode sizes in this paper are calculated by simulation, and the electric field simulation results are shown in Figure 7b and Figure 8b. The electric field nonuniform coefficient between the plate-plate electrodes is 1.1, and the electric field nonuniform coefficient between the needle-ball electrodes is 28.7. So the electric field generated by the customized container meets the test requirements. The detailed parameters of the electrode in the container are shown in Table 3, and the photographs of the test container are shown in Figure 7 and Figure 8c.

The test results show that burn marks will appear on the surface of the electrode after breakdown, and these marks and burn products will affect the electric field distribution between the electrodes. Therefore, the container designed in this paper uses the removable electrode structure and the burned electrode can be replaced easily, which can ensure the consistency of the test.

The needle-ball electrodes used in this paper are customized stylus and steel balls, and its electrode parameters and surface roughness meet the test requirements [30]. The plate-plate electrodes used in this paper are customized copper plates. In order to reduce the influence of electrode surface roughness on test results, the plate electrode in this paper is polished by 600, 1500, 3000, 5000, and 7000 mesh sandpaper step by step to have a mirror effect.

Before the test, the prepared electrode should be washed with pure water (without electrolyte), then washed with acetone (analytical reagent) and petroleum ether (analytical reagent, produced in Sinopharm Chemical, China), and finally dried in a vacuum drying oven for 2 h [28]. When drying, the temperature in the vacuum drying oven is 80 °C, and the air pressure is less than 5 kPa.

## 4. Methods and Procedures of the Withstand Voltage Test in Laboratory

In order to study the effect of cellulose particle content levels on the breakdown characteristics of transformer oil, it is necessary to apply power frequency voltage, lightning impulse voltage and oscillatory lightning impulse voltage to the four kinds of transformer oil samples. Therefore, a reasonable test procedure needs to be developed, which can ensure the orderly conduct of the test.

In this paper, the test procedure was developed according to relevant research and national standards “Insulating Liquids—Determination of the Breakdown Voltage at Power Frequency (GB/T 507-2002)”, “Methods for the Determination of the Lightning Impulse Breakdown Voltage of Insulating Liquids (GB/T 21222-2007)”, “Preventive Test Code for Electric Power Equipment (DL/T 596-2021)”, “Power Transformers—Part 3: Insulation Levels, Dielectric Tests and External Clearances in Air (GB/T 1094.3-2017)”, “Power Transformers—Part 4: Guide to the Lightning Impulse and Switching Impulse Testing—Power Transformers and Reactors (GB/T 1094.4-2005)” [28,29,30,31,32,33].

### 4.1. Preparation Before the Test

#### 4.1.1. The Sample Container and Electrodes

The sample container and electrodes should be washed with detergent and then rinsed twice with alcohol (analytical reagent) before testing. If there is still oil in the container, it needs to be washed with acetone (analytical reagent). Finally, the sample container and electrodes should be dried in a vacuum drying oven for 3 h. When drying, the temperature in the vacuum drying oven is 80 °C, and the air pressure is less than 5 kPa.

It is recommended to use a specific container for each transformer oil sample to ensure the reliability and stability of the test data.

#### 4.1.2. The Transformer Oil Sample

The packaged transformer oil sample should be stirred with a magnetic mixer for 5 min, and then dried in a vacuum drying oven for 48 h to remove the moisture. When drying, the temperature in the vacuum drying oven is 80 °C, and the air pressure is less than 5 kPa.

The transformer oil sample needs to be stirred with a magnetic mixer for 5 min before use, and the stirred sample should not have lumpy or cloud-like cellulose particles, such that the microcrystalline cellulose is evenly distributed in the transformer oil. After stirring, the transformer oil sample needs to stand for 5 min before the test, and the test time should be controlled within 3 min.

#### 4.1.3. The Test System

The transformer oil sample should be slowly poured into the unused container, and then the container should be closed.

For the power frequency voltage test, the sample and electrodes should be broken down 24 times by increasing the voltage. And for the lightning impulse voltage test, the sample and electrodes should be broken down 5 times by increasing the voltage.

After the breakdown, the container and electrodes will be infiltrated by the transformer oil sample, and the electrical components of the test system will have also completed various debugging procedures, so that the amplitude of the breakdown voltage during the test will be stable.

### 4.2. Power Frequency Voltage Test

Procedure 1

Pour out the transformer oil sample in the container, then slowly pour in the new transformer oil sample and close the container.

Turn on the magnetic mixer and stir the sample for 5 min, further homogenize the distribution of cellulose particles in the transformer oil, and accelerate the discharge of bubbles in the oil.

And the stirred transformer oil sample needs to stand for 5 min before starting the formal test.

Procedure 2

Slowly increase the voltage until the transformer oil sample is broken down, and record the breakdown voltage value. And the voltage increase rate between electrodes is 2 kV/s ± 0.2 kV/s.

Procedure 3

After the breakdown, stir the transformer oil sample for 3 min, and the sample needs to stand for 5 min before the next test.

After 6 repeated tests, the average breakdown voltage of 6 times is calculated as the test result.

During the test, it is necessary to ensure that there are no bubbles between the electrodes.

**Figure 9:**
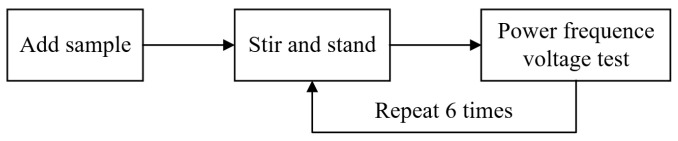
Power frequency voltage test procedure.

The simple test procedure is shown in Figure 9.

### 4.3. Lightning Impulse Voltage Test

Procedure 1

Pour out the transformer oil sample in the container, slowly pour in the new transformer oil sample and then close the container.

Turn on the magnetic mixer and stir the sample for 5 min, and the stirred sample needs to stand for 5 min before starting the formal test.

Procedure 2

The recommended voltage applied on the transformer oil sample is the standard lightning impulse voltage and the oscillating lightning impulse voltage with an oscillation frequency of 310 kHz.

For determining the 10% and 90% breakdown voltages, this paper uses the up-and-down method. And the effective voltage stages are 8, while the shocks per stage are 7.

For determining the breakdown voltage of other probabilities, this paper uses the multiple-stage method. And the voltage stages are not less than 6, while the shocks per stage are 30.

Procedure 3

During the test:

(a) If the transformer oil sample does not break down, stir the sample for 1 min, and then let stand for 2 min before the next test.

(b) If the transformer oil sample breaks down, stir the sample for 3 min, and then let stand for 5 min before the next test.

Procedure 4

In order to ensure that the test results are not affected by the electrode status and the transformer oil decomposition products, the following steps are taken:

(a) For the uniform electric field test, this paper replaces the transformer oil sample after 10 instances of breakdown, and replaces the plate-plate electrode after 20 instances of breakdown.

(b) For the uneven electric field test, this paper replaces the needle electrode and turns the ball electrode after 1 breakdown, and replaces the ball electrode and the transformer oil sample after 5 instances of breakdown.

**Figure 10:**
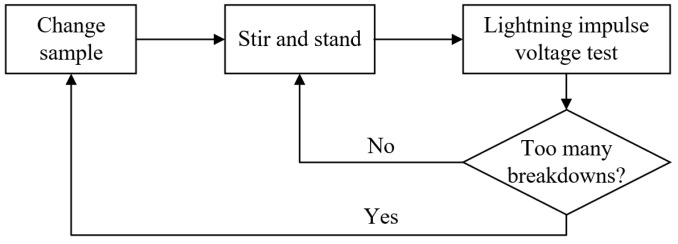
Lightning impulse voltage test procedure.

The simple test procedure is shown in Figure 10.

## 5. Conclusions

In this paper, a method for preparing four kinds of transformer oil samples with different cellulose particle content levels in the laboratory is provided, in which the KI-25X and the microcrystalline cellulose are selected as the material. And the physicochemical properties of the samples are close to the transformer oil in actual operation.

Based on engineering practice, this paper provides a laboratory impulse voltage test platform, which can generate lightning impulse voltage or oscillation lightning impulse voltage by replacing a small number of electrical components.

The laboratory impulse voltage test platform uses the plate-plate electrode to form the uniform electric field and uses the needle-ball electrode to form the uneven electric field. And the transformer oil sample container designed in this paper uses the removable electrode structure, so the burned electrode can be replaced easily.

Based on national standards and engineering practice, this paper puts forward a method of the withstand voltage test for transformer oil in the laboratory, which can improve the efficiency and the scientificity of such experiments. The withstand voltage test method in this paper is applicable to the voltage grade below 500 kV, and the circuit and electrode parameters mentioned in this paper are applicable to experiments below 100 kV.

## Figures and Tables

**Table 1:** Quality parameters of the transformer oil sample after pretreatment.

Test Item	Test Result	Quality Indicator	Voltage Grade
Moisture (mg/L)	4.3	≤10	500 kV
Breakdown voltage (kV)	65.4	≥65	500 kV
Particulate contamination (grain/100 mL)	1527	≤2000	500 kV
Dielectric loss factor	0.001	≤0.005	500 kV
Acid value (mg/g)	0.006	≤0.01	750 kV
Furfural content (mg/kg)	0.01	≤0.05	750 kV

**Table 2:** Circuit parameters of the test platform.

Output Voltage Waveform	Standard Lightning Impulse Voltage	Oscillation Lightning Impulse Voltage
R (MΩ)	2.67	2.67
C_1_ (nF)	10.25	10.25
C_2_ (nF)	9.68	9.68
R_f_ (kΩ)	0.99	0.005
R_t_ (kΩ)	6.38	39.96
L (mH)	0.00	0.62

**Table 3:** Parameters of the electrode in the container.

Electric field type	Uniform	Uneven
	electric field	electric field
Electrode size	Plate thickness	Needle curvature
	3.9 mm	65 μm
	Plate diameter	Ball diameter
	24.1 mm	12.5 mm
Electrode gap	1 mm ± 0.02 mm	5 mm ± 0.1 mm
Surface roughness	<6 μm	<6 μm
Nonuniform coefficient	1.1	28.7

## Data Availability

The data presented in this study are available on request from the corresponding author. The data are not publicly available due to privacy limitations.
